# Management of Spontaneous Bleeding in COVID-19 Inpatients: Is Embolization Always Needed?

**DOI:** 10.3390/jcm10184119

**Published:** 2021-09-12

**Authors:** Pascale Riu, Fabrizio Albarello, Federica Di Stefano, Alessandra Vergori, Alessandra D’Abramo, Carlo Cerini, Martina Nocioni, Maurizio Morucci, Nardi Tetaj, Massimo Cristofaro, Vincenzo Schininà, Paolo Campioni, Ada Petrone, Nicoletta Fusco, Luisa Marchioni, Andrea Antinori, Emanuele Nicastri, Roberto Cianni, Stefania Ianniello

**Affiliations:** 1 Interventional Radiologist Unit, Azienda Ospedaliera San Camillo Forlanini, 00149 Rome, Italy; priu@scamilloforlanini.rm.it (P.R.); mmorucci@scamilloforlanini.rm.it (M.M.); rcianni@scamilloforlanini.rm.it (R.C.); 2 Radiology Unit, National Institute for Infectious Diseases Lazzaro Spallanzani IRCCS, 00149 Rome, Italy; federica.distefano@inmi.it (F.D.S.); massimo.cristofaro@inmi.it (M.C.); vincenzo.schinina@inmi.it (V.S.); paolo.campioni@inmi.it (P.C.); ada.petrone@inmi.it (A.P.); nicoletta.fusco@inmi.it (N.F.); stefania.ianniello@inmi.it (S.I.); 3 HIV/AIDS Unit, National Institute for Infectious Diseases Lazzaro Spallanzani IRCCS, 00149 Rome, Italy; alessandra.vergori@inmi.it (A.V.); carlo.cerini@inmi.it (C.C.); andrea.antinori@inmi.it (A.A.); 4 Emerging Infectious Diseases Unit, National Institute for Infectious Diseases Lazzaro Spallanzani IRCCS, 00149 Rome, Italy; alessandra.dabramo@inmi.it (A.D.); emanuele.nicastri@inmi.it (E.N.); 5 Intensive Care Unit, National Institute for Infectious Diseases Lazzaro Spallanzani IRCCS, 00149 Rome, Italy; martina.nocioni@inmi.it (M.N.); nardi.tetaj@inmi.it (N.T.); luisa.marchioni@inmi.it (L.M.)

**Keywords:** COVID-19, spontaneous bleeding, percutaneous trans arterial embolization, low-molecular-weight heparin

## Abstract

Background: critically ill patients with SARS-CoV-2 infection present a hypercoagulable condition. Anticoagulant therapy is currently recommended to reduce thrombotic risk, leading to potentially severe complications like spontaneous bleeding (SB). Percutaneous transcatheter arterial embolization (PTAE) can be life-saving in critical patients, in addition to medical therapy. We report a major COVID-19 Italian Research Hospital experience during the pandemic, with particular focus on indications and technique of embolization. Methods: We retrospectively included all subjects with SB and with a microbiologically confirmed SARS-CoV-2 infection, over one year of pandemic, selecting two different groups: (a) patients treated with PTAE and medical therapy; (b) patients treated only with medical therapy. Computed tomography (CT) scan findings, clinical conditions, and biological findings were collected. Results: 21/1075 patients presented soft tissue SB with an incidence of 1.95%. 10/21 patients were treated with PTAE and medical therapy with a 30-days survival of 70%. Arterial blush, contrast late enhancement, and dimensions at CT scan were found discriminating for the embolization (*p* < 0.05). Conclusions: PTAE is an important tool in severely ill, bleeding COVID-19 patients. The decision for PTAE of COVID-19 patients must be carefully weighted with particular attention paid to the clinical and biological condition, hematoma location and volume.

## 1. Introduction

Since December, 2019, Wuhan, China, has experienced an outbreak of coronavirus disease 2019 (COVID-19), caused by the severe acute respiratory syndrome coronavirus 2 (SARS-CoV-2) [1]. 

After one year of the pandemic, the scientific community has recognized COVID-19 to have a multisystemic involvement, most frequently related with a severe acute respiratory syndrome, but also presenting, less frequently, gastrointestinal (GI) manifestations and, rarely, neurological complications [2,3]. Moreover, all critical patients with COVID-19 showed a higher risk of evolving a hypercoagulable condition [4]. 

Coagulopathy results from haemostatic changes that might be a direct effect of SARS-CoV-2 or a consequence of a severe pro-inflammatory state (i.e., the so-called cytokine storm) that amends the approach of the systemic inflammatory response syndrome (SIRS) [5].

With the purpose to contain the increased incidence of pulmonary embolism (PE) reported, an anticoagulation therapy was approved for the treatment of COVID-19 [6]. It is advisable, indeed, to offer prophylactic anticoagulation with low-molecular-weight heparin (LMWH) as early as possible to prevent thrombotic events and organ damage, as recommended by the International Society on Thrombosis and Haemostasis (ISTH) guidance on the detection and treatment of coagulopathy in COVID-19 [7].

In the meantime, an increased incidence of spontaneous bleeding (SB) was observed [8,9,10]. 

Interventional radiology management with Percutaneous Trans Arterial Embolization (PTAE) of patients with SB is not codified by international guidelines and is usually assessed on a single basis and offered to patients with hemodynamic instability or failure of medical management [11].

Here, we report 21 cases of soft tissue SB found during a 4-phase computed tomography (CT) scan in patients with severe COVID-19 pneumonia, hospitalized at the National Institute for Infectious Diseases (INMI), L. Spallanzani, IRCCS, Rome, Italy, the main regional infectious disease centre. Ten patients underwent PTAE, whilst 11 were treated conservatively, based on the local protocol.

Clinical and radiological features within the two groups of patients were compared in order to find a possible selection criterion for the most successful approach. 

## 2. Materials and Methods

### 2.1. Study Population and Setting

We retrospectively considered all patients with SB of different anatomical regions and with a microbiologically confirmed SARS-CoV-2 infection who were hospitalized at the INMI, between March 2020 and March 2021. Consequently, we classified two different groups: (a) patients treated with PTAE and medical therapy; (b) patients treated only with medical therapy in a conservative manner.

### 2.2. Definitions

A confirmed case of COVID-19 was defined by a positive real-time reverse-transcription PCR (RT-PCR) assay for SARS-CoV-2 on a nasopharyngeal swab and/or a positive serology for SARS-CoV-2 (positive Immunoglobulin (Ig) G or M or A for SARS-CoV-2). Severe disease was defined as clinical signs of pneumonia plus one of the following: respiratory rate greater than 30 breaths per min, severe respiratory distress, or oxygen saturation less than 90% in room air [12]. Hyperinflammation syndrome was defined as having at least two of the following: D-dimer above 1000 ng/mL, ferritin above 500 mcg/L, LDH above 300 UI/L, and lymphocyte count below 1000 cell/mm3 [4]. SB was diagnosed by using a CT scan. The estimated glomerular filtration rate (eGFR) was calculated by CKD-EPI formula. Low molecular weight heparin anticoagulant prophylaxis (LMWH) was administered to all patients at a dosage of 100 UI/Kg/day, according to local protocol, on the day of the hospital admission. Only one patient was taking an anti-platelet agent (acetylsalicylic acid) together with LMWH prophylaxis. All the patients selected for embolization were transferred to the nearest hospital with an interventional radiology unit and underwent PTAE.

### 2.3. CT Examination

All 21 patients underwent a 4-phase CT examination including an unenhanced CT, an arterial phase with bolus detection software, a venous phase at 70–90 s, and a late phase at 180 s. All CT scans were carried out on a multi-detector row CT scanner (Bright Speed, General Electric Medical Systems, Milwaukee, WI, USA) by using 120 kV pp, 250 mA, pitch of 1.375, and gantry rotation time of 0.6 s. A thorax and abdomen CT were carried out from the apex of lung to symphysis pubis before and after injection of iodinated contrast media into a peripheral vein. The baseline scan of the chest was reconstructed with slice thicknesses of 0.625 mm and spacing of 1 mm with a high contrast resolution algorithm. The contrast media scan of the thorax and abdomen were reconstructed with slice thicknesses of 1.25 mm and spacing of 1 mm, complete with multiplanar reconstructions (MPR and Mip). Results of the CT examinations were retrospectively analysed by two radiologists (SI and FdS with 15 and 10 years of experience in abdominal imaging) and a team of interventional radiologists (with more than 15 years of experience in PTAE) working in consensus. The location of the hematoma was noted, and active bleeding was defined as the presence of contrast material extravasation that appeared on arterial phase CT images and grew on venous and late-phase images. The bleeding localization, intra or extra-muscular involvement, and the dimensions of the hematoma (considered as the sum of the two major diameters on the three planes ÷ 2) were collected.

### 2.4. PTAE

All patient candidates for PTAE were transferred to the nearest hospital with interventional radiology available for immediate treatment, and an operating room dedicated to COVID-19 patients, equipped with a C-arm (Ziehm-imaging GmbH, Nuremberg, Germany). All procedures were performed by an experienced interventional radiologist (with more than 15 year of experience). The right common femoral artery was accessed with a 5-FR sheath introducer. The vessel responsible for the current bleeding, previously identified with a CT scan, was catheterized with a 5 FR catheter (Simmons/Sidewinder 1, Multipurpose, Medikit, Tokio, Japan), then with a microcatheter (Terumo 2.7 FR, Terumo Corporation, Tokio, Japan) when needed and then embolized. All the arteries of the same anatomic territory were embolized to avoid rebleeding due to anastomotic reperfusion. The embolization was performed with particles (PVA contour 350–500/500–700 um) +/− micro coils +/− gel foam. In one patient glue mixture, 1:3 of n-BCA (n-butyl cyano-acrylate) and lipiodol (Ultra Fluid-Guerbet, Paris, France) were used. The embolization was performed empirically even without sign of active bleeding during the procedure. The final angiogram confirmed in all cases a good morphological result with no more evidence of bleeding lesions. The decision for PTAE was based upon the clinical scenario of the patients: (i) always done in hemodynamically unstable patients, (ii) based on different severity criteria in hemodynamically stable. Severity criteria included: (a) active bleeding at CT, (b) large hematomas, (c) retroperitoneal haemorrhage, (d) rupture of the hematoma’s wall with intra or peritoneal haemorrhage, (e) risk factors as dropping of haemoglobin (>2–3 g/dL) and/or need of repeated transfusions, COVID-19 related severity and complications (pulmonary embolism, difficulty to retrieve anti-coagulation). Failure of conservative management alone was an indication too.

### 2.5. Outcomes

Technical success was defined as successful catheterization and embolization of the artery of the territory, whilst clinical success as the hemodynamic and haemoglobin stabilization of the patient at 3 h since the embolization. Overall survival was evaluated within 30 days.

### 2.6. Statistical Analysis

Quantitative parameters and continuous data are presented as median and interquartile range (IQR, 25th–75th percentile) and were compared between groups using the parametric T-student test. Qualitative parameters are presented as counts and percentage. Categorical variables were compared using the Fischer’s exact test. Statistical significance was set at *p* < 0.05. All statistical analyses were carried out with SPSS (IBM Corporation, Chicago, IL, USA) Results version 20.0. 

## 3. Results

A total of 1075 adult patients who tested positive for SARS-CoV-2 were admitted to our institution during the study period.

### 3.1. COVID-Related Clinical Characteristics 

Severe clinical presentation was noted in 10 patients with an admission oxygen saturation (SpO2) 93% on room air (IQR 90–98). The median arterial oxygen partial pressure (PaO2 in mmHg) to fractional inspired oxygen (PaO2/FiO2) ratio was 200 mmHg (103–273) and it required in eight patients supplemental oxygen therapy with non-invasive ventilation. Seven subjects required invasive mechanical ventilation with orotracheal intubation (OTI) and admission to the ICU. 

All patients received prophylactic low weight molecular heparin (LWMH) at admission and the heparin dosages were modified based on clinical deterioration and for the diagnosis of micro or macro pulmonary thromboembolic events. Overall, in 20–21 patients (95.2%), anticoagulant dosage was prescribed during the hospitalization. 

Overall, steroid therapy was dispensed to 18 patients (85.7%). Specifically, with regards to SARS-CoV-2 therapy, oral lopinavir/ritonavir (LPV/r, 400/100 mg twice per day for 14 days) were administered to 3 patients, one of them even received oral hydroxychloroquine (200 mg twice per day for 10 days); intravenous Remdesivir (200 mg on day one followed by 100 mg since day 2 to day 10) (52.6%) was administered to 10 patients. Inflammation and coagulation parameters are displayed under Table 1—any patient showing a hyperinflammation pattern with a median ferritin level of 541 pg/mL (IQR 297–1113) and C-reactive protein (CRP) of 6.6 mg/dL (IQR 4.5–16.0) and a D-dimer of 740 ng/mL (IQR 555–1473). 

No statistical differences were observed in all these characteristics, other than a greater lymphopenia (1200 vs. 605 cells × 1000/mcL, *p* = 0.003). The median hospitalization lasted 34 days (IQR 24–51) in all cohort, reaching 37 days if considering the surviving patients. 

### 3.2. Radiological Characteristics and Interventional Outcomes

All the patients presented pneumonia, and chest CT scan performed at admission revealed bilateral ground–glass opacities (GGOs) and sub-segmental consolidations, largely located in the peripheral zone. Moreover, other imaging characteristics such as linear opacities, “crazy-paving” pattern, the “reverse halo sign”, as well as subsegmental vessel enlargement were described. More specifically, the vessel enlargement was illustrated close to the GGOs, which is compatible with thrombo-inflammatory processes. After contrast media CT scan, we observed in nine subjects thrombosis-mediated micro-perfusion defects in peripheral pulmonary vessels (42.8%) within the included patients.

- 21/1075 (1.95%) COVID-19 patients with haemorrhagic events were observed.
- Eleven patients were treated conservatively, based on clinical judgement, resuscitated with intravenous fluid, transfusions of red blood cells and, in addiction, further standard supportive measures.
- Ten patients, in addition to the conservative treatment, underwent PTAE after the haemorrhagic event.
- Three patients treated with PTAE died after the event within 30 days (14% of lethality) whereas one patient died after more than 90 days of the procedure for independent reasons.
- Patients treated with a conservative manner only had a 30-day survival of 90.9%. A deceased patient in this group presented terminal complication of recent major hepatic surgery, and was not considered for PTAE.

Radiological and interventional features as well as the drop in haemoglobin and short survival after the procedure within the two groups of patients (embolized and not-embolized) are summarized in Table 2.

**Figure 1:**
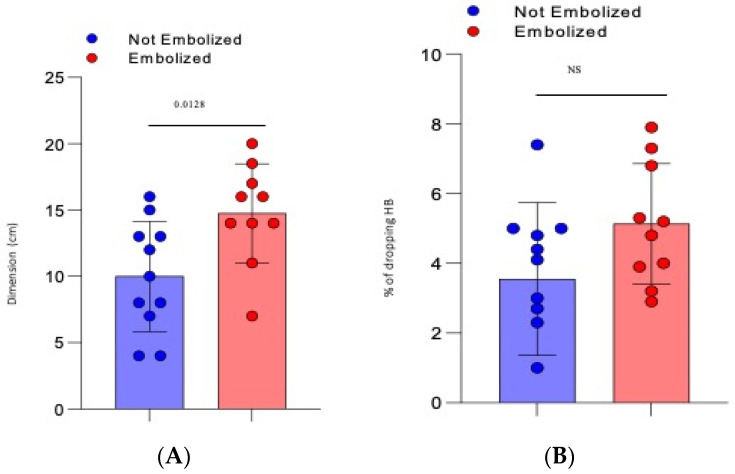
Severity criteria within the two groups: (**A**) Dimension of the hematoma (considered as the sum of the two major diameters on the three planes ÷ 2); (**B**) % of dropping of hemoglobin (>2–3 g/dL).

**Figure 2:**
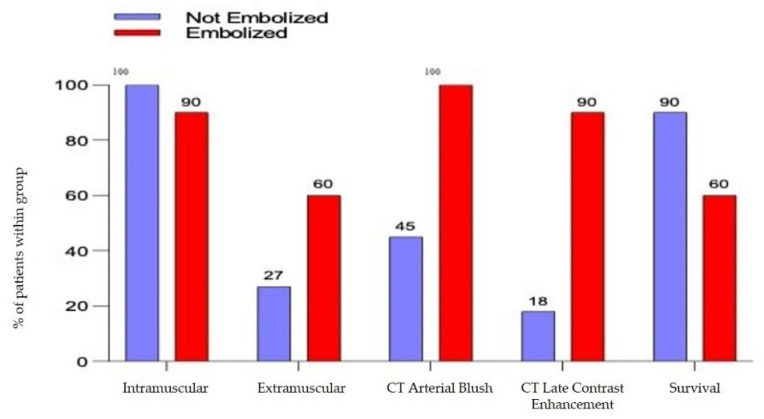
Severity criteria detected with computed tomography and overall survival.

- The mean dimension of the hematoma was 10 cm (±1.25 SD) for the not-embolized group vs. 14.75 cm (±1.2 SD) for the embolized group (*p* = 0.0128), Figure 1A.
- There was a mean drop in haemoglobin of 3618 g/dL (±0.622 SD) for the not-embolized group vs. 5.13 g/dL (±0.55 SD) for the embolized group (*p* = 0.086), Figure 1B.
- The arterial blush at CT scan was presented in all the embolized patients but only in 5 patients not embolized (45%) with a significative difference (*p*= 0.0124). There was a significant difference also in the presence of a CT Late Contrast Enhancement between the 2 groups (90% vs. 18,1%, embolized vs. not embolized, *p* = 0.0019), Figure 2.
- The presence of an intra or extra-muscular hematoma was not statistically significant (Figure 2).

### 3.3. Outcomes

Technical success was achieved in all the embolized patients and clinical success in 9/10 (90%); 1 patient presented haemodynamic instability and died the same day of the PTAE. Overall survival at 30 days was reported in 7/10 patients (70%). Two patients required a second PTAE for recurrent bleeding with a full recovery. No short-term complications were observed after PTAE.

## 4. Discussion

SB of soft tissue is defined as an intra or extra-muscular collection of blood, mainly located in the ilio-psoas and rectus muscle. It is a potentially severe complication for patients under anticoagulant treatment that can lead to hemodynamic instability and is therefore life-threatening [13,14]. French authors reported a mortality rate as high as 30% occurring in the ilio-psoas, more frequently in ICU [15], with an incidence in the literature ranging from 0.1 to of 0.6% [16]. 

We report an incidence of 1.95 cases over 100 hospitalizations for COVID-19 patients, under anticoagulation therapy, higher than that previously reported. An explanation could be found in the particular physiopathology of SARS-CoV-2 infection related to endothelial damage [17,18]. Risk factors gathered in our small case series are equal to that reported in literature, such as age, anticoagulation therapy, high body mass index, and comorbidities such as hypertension and diabetes [19,20,21,22]. 

The clinical management of haemorrhage is classically based on correction of coagulation parameter, discontinuation of anticoagulant agents, volume fluid resuscitation, transfusion therapy, and supportive procedures; haemodynamically unstable patients, according to expert clinical judgement, can be treated with PTAE, due to its minimally invasive and quick therapeutic effect, compared to surgical treatment [15,23,24,25]. PTAE is a well-accepted, safe, and effective treatment in many other clinical settings, such as polytrauma, post-partum haemorrhage, or haemoptysis [26,27]. Nevertheless, there is still a lack of treatment guidelines on arterial embolization in SB [24,25]. To date, the treatment indication and predictors of mortality have not been well identified. 

The volume of hematoma detected at CT, often with worse clinical conditions, has been the most significant factor for a PTAE choice. The dimension was also predictive of mortality, as all the deceased patients had large hematomas. Moreover, we found a poor outcome among the embolized hematoma with a retroperitoneal location, as already reported by Barral et al. [28]. Despite the COVID-19 inpatients fitting all the criteria for a bad clinical scenario (more often elderly people, with at least 2–3 comorbidities and obese), the mortality found in the embolized group was similar to other series reported in different clinical settings [25,28]. Furthermore, in all patients, embolization was technically successful. Only one patient died directly because of haemodynamic instability on the same day, as a clinical failure.

Among the others embolized patients who died, we were not always able to clearly distinguish between the responsibility of COVID-19 and the consequences of haemorrhage by the delayed worsening of general conditions; nevertheless, we observed a greater lymphopenia in this group, as a sign of severity of the infection.

The precise physiopathology of SB in anticoagulated patients is unknown. The vessel fragility is heightened by micro-atherosclerosis, with fat involution of the muscle in the elderly, leading to muscular tears and consequential bleeding. Mobilisation of the patient in the ICU, the coughing, the vomiting, or the breathing exercise with pronation, part of the therapy of COVID 19, as muscle isometric effort, can also lead to micro trauma and bleeding [15,29,30,31]. The SARS-CoV-2 infection carries a special condition of endothelitis due to both viral infection of the cell and inflammatory response, with the consequence of micro and macro thrombosis, and also endothelial injury leading to micro vessels’ fragility [17]. This vascular condition associated with micro and macro thrombosis is the very reason to treat these patients aggressively with heparin, with a consequent difficulty to safely retrieve anti-coagulation treatment when needed.

**Figure 3:**
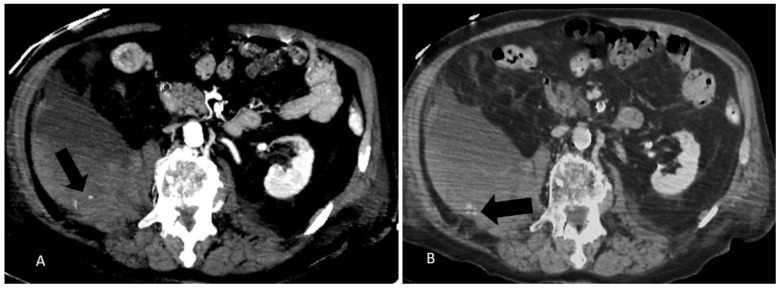
(**A**) Arterial phase CT and (**B**) late phase CT of a right retroperitoneal and ileo-psoas hematoma. Note multiple arterial intralesional blushes (arrow in (**A**)) show a contrast late enhancement (arrow in (**B**)).

**Figure 4:**
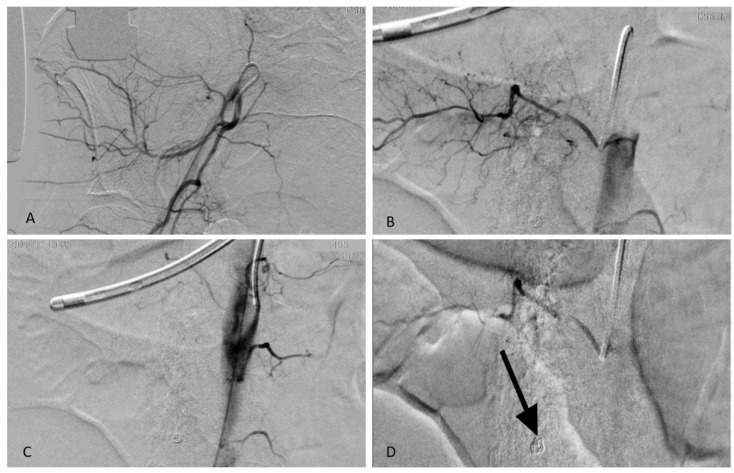
(**A**) Catheterism of the right hypogastric artery to reach the right ileo-lumbar artery (anastomotic territory); (**B**) Lumbar artery catheterization showing tiny bleeding spots; (**C**) Post-embolization angiogram: right lumbar arteries occluded; (**D**) Stop flow post embolization with particles (PVA contour 350–500) in the 4th lumbar artery and coil in the 5th lumbar artery (arrow).

During a CT scan, active bleeding is a warning sign, and a call for urgent management. Arterial blush and late contrast enhancement have to be considered during the decision to treat, due to the high sensitivity of the angio-CT (87%). It also represents a really useful tool to guide embolization as spasm, hypotension, tamponade, and technical limits can restrain the visibility of the bleeding at angiography (Figure 3 and Figure 4); however, PTAE should be performed in severe clinical bleeding even without the sign of active bleeding at CT or angiography as the negative predictive value is low [25].

Unlike some authors [10], according to our experience, the selected stable patients can be managed medically and conservatively, as shown by the survival results of the non-embolized group. Since no consensus on therapeutic management of SB has been achieved, any choice (conservative treatment or embolization) should be ultimately made by the clinical physician (along with the help of interventional radiologists) in accordance with the clinical status of patients and doing a trade-off between risk and benefit factors.

About the technique, a definitive embolic (particles, coils and glue) seems to be more effective, as we observed no death versus three with a temporary embolic [32].

The interventional radiologist must be aware of technical difficulties during PTAE: the arteries are more fragile and susceptible to dissection and coils can be difficult to be released, due to vascular spasms. We definitively prefer particles as they better fit the very distal injury of micro-vessels in anticoagulated and COVID-19 patients. Liquid embolics like glue seems to be a good option too. Furthermore, the embolization can be done more proximally, avoiding delicate catheter navigation in fragile and injured vessels.

Our study has several limitations: the results are based on a retrospective analysis of radiologic and clinical data of a single reference centre. The final decision to treat was taken by the senior physician on duty, on a clinical basis. In addition, in this COVID-19 pandemic, physicians are faced with several logistic difficulties in the management of these patients, such as the difficult transport and relocation of patients in a dedicated operating room, with limited technology (mobile C-Arm), in order to avoid further spread of the infection in non-COVID-19 facilities and this may have slightly impacted or delayed treatment.

Among the patients who died, we were not able to clearly distinguish between responsibility of COVID-19 and the consequences of haemorrhage caused by the delayed worsening of general conditions.

## 5. Conclusions 

PTAE is an important tool in the treatment of SB in severely bleeding COVID-19 patients under anticoagulant therapy. However, this serious condition can be fatal, despite treatment. A group of well-selected, less-serious bleeding patients can be managed expectantly, with good results with standard medical management. 

In our opinion, the indications of PTAE, the timing, and technique should be well defined when dealing with COVID-19 patients, given the very specificity of the disease. The decision to embolize must be done carefully with particular attention paid to clinical conditions (hemodynamic/dropping haemoglobin and hematoma volume).

In conclusion, the optimal dose and duration of anti-coagulations has to be monitored, being aware of potentially serious complication in COVID-19 inpatients.

## Figures and Tables

**Table 1:** Sociodemographic, laboratory tests, and clinical characteristics.

*N* (%), Median (IQR)	Overall	Not Embolized *N* = 11	Embolized *N* = 10	*p*-Value
Gender				
Male	13 (61.9%)	5 (45.5%)	8 (80%)	0.104
Female	8 (38.1%)	6 (54.5%)	2 (20)	
Age, years	66 (62–76)	64 (56–74)	75 (62–80)	0.573
BMI	27 (25–29)	28 (23–29)	25 (25–29)	0.906
*N*. of co-morbidities				0.086
0	2 (9.5%)	2 (18.2%)	0	
1	6 (28.6%)	2 (18.2%)	4 (40%)	
2	6 (28.6%)	5 (45.5%)	1 (10%)	
3+	7 (33.3%)	2 (18.2%)	5 (50%)	
CVD co-morbidity	15 (71.4%)	8 (72.7%)	7 (70%)	0.890
DM co-morbidity	4 (19.1%)	3 (27.3%)	1 (10%)	0.314
PaO_2_/FiO_2_ at admission < 300 mmHg	200 (103–273)	200 (103–251)	215 (123–292)	0.724
Ferritin, pg/mL	541 (297–1113)	521 (336–831)	707 (248–1412)	0.790
CRP, mg/dL	6.6 (4.5–16.0)	8.6 (3.5–16.2)	6.2 (4.8–16.0)	0.725
LDH, U/L	328 (277–404)	325 (254–433)	356 (309–404)	0.291
D-dimer, ng/mL	740 (555–1473)	879 (555–1496)	664 (403–1473)	0.481
Lymphocytes, cells × 1000/mcL	960 (640–2650)	1200 (960–7500)	605 (510–760)	0.003
D-dimer, ng/mL				0.639
<500	4 (19.1%)	1 (9.1%)	3 (30%)	
501–1000	10 (47.5%)	6 (54.5%)	4 (40%)	
1000–2500	4 (19.1%)	2 (18.2%)	2 (2.0%)	
>2500	3 (14.3%)	2 (18.2%)	1 (10%)	
Antiviral therapy				0.620
LPV/r	2 (9.5%)	1 (9.1%)	1 (10%)	
HCQ	0	0	0	
LPV/r+HCQ	1 (4.8%)	1 (9.1%)	0	
Neither LPV nor HCQ	18 (85.7%)	9 (81.8%)	9 (90%)	
Steroides	18 (85.7%)	9 (81.8%)	9 (90%)	0.228
Remdesivir	10 (52.6%)	6 (54.6%)	4 (50%)	0.845
Hospitalization, days	34 (24–51)	37 (29–50)	26 (17–79)	0.409
Days from symptoms onset to Hematoma	25 (14–35)	25 (15–33)	30 (13–37)	0.790
Intramuscular Hematoma	20 (95.2%)	11 (100.0%)	9 (90%)	0.283
Deaths	5 (23.8%)	1 (9.1%)	4 (40%)	0.097

Lactic dehydrogenase (LDH), C reactive protein (CRP), Diabetes mellitus (DM), Cardiovascular disease (CVD), Lopinavir/ritonavir (LPV/r), Hydroxychloroquine (HCQ), body mass index (BMI).

**Table 2:** Major radiological features and severity criteria within embolized and not-embolized patients.

	Overall	Not Embolized *N* = 11	Embolized *N* = 10	*p*-Value
Dropping HB	4.3	3.55	5.13	0.084
Bleeding localization				0.598
Left iliopsoas	6 (28.5%)	4 (36.3%)	2 (20%)	
Right iliopsoas	5 (23.8%)	4 (36.3%)	1 (10%)	
Abductor muscles	1 (4.7%)	1 (9.09%)	0 (0%)	
Paravertebral muscles	1 (4.7%)	1 (9.09%)	0 (0%)	
Thoracic muscles	2 (9.5%)	0 (0%)	2 (20%)	
Rectus abdominis	3 (14.2%)	1 (9.09%)	2 (20%)	
Gluteus	1 (4.7%)	0 (0%)	1 (10%)	
Retropancreatic	1 (4.7%)	0 (0%)	1 (10%)	
Left pectineus	1 (4.7%)	0 (0%)	1 (10%)	
Intramuscular	20 (95.2%)	11 (100%)	9 (90%)	NS
Extramuscular	9 (42.8%)	3 (27.3%)	6 (60%)	NS
Dimension (cm)	12.26	10	14.75	0.0128 *
CT Arterial Blush	15 (71.4%)	5 (45.4%)	10 (100%)	0.0124 *
CT Late CE	11 (52.4%)	2 (18.2%)	9 (90%)	0.0019 *
Outcome (survival)	16 (76.2%)	10 (90.9%)	6 (60%)	NS

* Statistical difference, Haemoglobin (B), Contrast enhancement (CE), Not significative (NS).

## Data Availability

Data was collected for the ReCOVeRI Study, a registry on COVID-19 for clinical Research of the National Institute for Infectious Diseases L. Spallanzani.
